# Bone Marrow-Derived Cell Therapies to Heal Long-Bone Nonunions: A Systematic Review and Meta-Analysis—Which Is the Best Available Treatment?

**DOI:** 10.1155/2019/3715964

**Published:** 2019-12-27

**Authors:** Silvia Palombella, Silvia Lopa, Silvia Gianola, Luigi Zagra, Matteo Moretti, Arianna B. Lovati

**Affiliations:** ^1^Cell and Tissue Engineering Laboratory, IRCCS Istituto Ortopedico Galeazzi, Milan 20161, Italy; ^2^Unit of Clinical Epidemiology, IRCCS Istituto Ortopedico Galeazzi, Milan 20161, Italy; ^3^Hip Department, IRCCS Istituto Ortopedico Galeazzi, Milan 20161, Italy; ^4^Regenerative Medicine Technologies Lab, Ente Ospedaliero Cantonale, Lugano 6900, Switzerland

## Abstract

Nonunions represent one of the major indications for clinical settings with stem cell-based therapies. The objective of this research was to systematically assess the current evidence for the efficacy of bone marrow-derived cell-based approaches associated or not with bone scaffolds for the treatment of nonunions. We searched MEDLINE (PubMed) and CENTRAL up to July 2019 for clinical studies focused on the use of cell-based therapies and bone marrow derivatives to treat bone nonunions. Three investigators independently extracted the data and appraised the risk of bias. We analysed 27 studies including a total number of 347 participants exposed to four interventions: bone marrow concentrate (BMAC), BMAC combined with scaffold (BMAC/Scaffold), bone marrow-derived mesenchymal stromal cells (BMSCs), and BMSC combined with scaffold (BMSC/Scaffold). Two controlled studies showed a positive trend in bone healing in favour of BMAC/Scaffold or BMSC/Scaffold treatment against bone autograft, although the difference was not statistically significant (RR 0.11, 95% CI -0.05; 0.28). Among single cohort studies, the highest mean pooled proportion of healing rate was reported for BMAC (77%; 95% CI 63%-89%; 107 cases, *n* = 8) and BMAC/Scaffold treatments with (71%; 95% CI 50%-89%; 117 cases, *n* = 8) at 6 months of follow-up. At 12 months of follow-up, an increasing proportion of bone healing was observed in all the treatment groups, ranging from 81% to 100%. These results indicate that BMAC or BMAC/Scaffold might be considered as the primary choice to treat nonunions with a successful healing rate at a midterm follow-up. Moreover, this meta-analysis highlighted that the presence of a scaffold positively influences the healing rate at a long-term follow-up. More case-control studies are still needed to support the clinical improvement of cell-based therapies against autografts, up to now considered as the gold standard for the treatment of nonunions.

## 1. Introduction

Nonunions and delayed unions are a frequent occurrence in fracture healing, with an incidence of 5-10% over the total amount of fractures only in the US [1]. Per definition, if a fracture does not heal within 4 months, it can be considered as a delayed union. Fractures of long bones—mainly the femur, tibia, and humerus—are defined as nonunions after 6 months postinjury in the absence of any radiographic progression persisting for at least 3 months [2, 3]. The poor healing of bone fractures is due to multiple factors, including patient-independent factors specifically related to the fracture site, severity of the injury (large gap), impaired vascularization, surgical osteosynthesis, infections, fracture stability, and biomechanics, and patient-dependent factors, such as age, nutrition, drug therapies, and comorbidities or congenital bone disorders [4, 5]. The diagnosis of nonunions is based mainly on clinical evaluation and radiographic imaging. The physical examination assesses the mobilization of the fracture site, deformity, interpolation of soft tissues, and signs of infection [6, 7]. X-rays are used to determine the progress of fracture healing (i.e., persistent fracture lines, absence of bony bridging, and sclerotic tissue) and the presence or absence of deformity [6]. Radiologically, nonunions can be distinguished as hypertrophic and atrophic. Hypertrophic nonunions are characterized by a large, broad callus towards the fracture gap, with a radiolucent area instead of bone bridging. On the other hand, in most cases, atrophic nonunions are the expression of an impaired biological support to bone healing, which may depend on a damaged vascular supply, and on the destruction of the periosteum and endosteum. Subsequently, the healing process is impaired due to the lack of important biological mediators and/or appropriate blood supply. Mechanical reasons can also be involved. For instance, excessively rigid fixation, insufficient compressive forces, and a wide fracture gap that does not allow bony bridging are major determinants in the development of atrophic nonunions. In radiological images, atrophic nonunions show the absence of callus tissue, the narrowing of bone ends, and a large radiolucent zone in the fracture gap [3]. For all these reasons, atrophic nonunions represent the most challenging occurrence. In fact, atrophic nonunions always require a surgical approach to reduce abnormal mechanical factors and repair the fracture gap by means of bone grafting, which represents the therapeutic gold standard [8].

Since both autograft and allograft have intrinsic limitations, such as the volume of collectable autologous bone and patient morbidity, or immunogenic rejection and risk of disease transmission, respectively, the use of orthobiologics is on the rise in translational medicine for bone repair [8]. Indeed, orthobiologics for bone healing implement the “diamond concept” combining osteoconduction, osteoinduction, and osteogenesis and, hence, appear as a promising strategy to treat nonhealing fractures, in particular atrophic nonunions [9]. Among orthobiologic approaches for bone healing, cell-based therapies, and bone marrow derivatives combined or not with bone grafts and biomaterials have been widely investigated in the recent years [10].

In orthopaedics, iliac crest aspiration is a standard procedure associated with limited morbidity for the harvesting of bone marrow, the most used source of adult mesenchymal stromal cells. Bone marrow can be used either directly as bone marrow aspirate concentrate (BMAC) or as bone marrow-derived mesenchymal stromal cells (BMSCs) after *in vitro* processing. Advantages of BMAC are the intraoperative preparation using CE-marked kits and centrifuges and compliance with a one-step surgery with limited costs [3]. Despite these benefits, BMAC accounts only for a small population of progenitor cells (0.001% to 0.01%), as compared to expanded BMSCs that are a pure cell population with well-defined features. However, in the clinical setting, the translatability of BMSC is still limited by some drawbacks, such as the need to use GMP-compliant growth factors and reagents and the extensive timeframe required for *in vitro* expansion in specialized facilities, which imply higher operational complexity and superior costs compared to BMAC.

This systematic review focuses on clinical studies regarding the use of bone marrow derivatives to treat nonunions. Specifically, we aimed to assess the efficacy of cell-based therapies with respect to bone autograft. In addition, we analysed the proportion of the healing rate at 6 and 12 months after treatment with BMAC or BMSC alone or combined with scaffolds. The aim of this meta-analysis is to increase the knowledge in the options available to promote bone healing and represent a tool for clinicians who plan to use cell-based strategies to improve the clinical outcomes of patients with nonunions.

## 2. Materials and Methods

### 2.1. Search Strategy

The literature search was focused on the clinical use of bone marrow-derived cell therapies to treat bone nonunions. The literature search was carried out consulting MEDLINE (PubMed) and Cochrane Central Register of Controlled Trials-CENTRAL databases, analysing articles published in English up to July 2019. We also checked the reference lists of all the systematic reviews and included studies identified during the search process. The full search strategy is reported in [Supplementary-material supplementary-material-1] in the Supplementary Materials.

### 2.2. Eligibility Criteria for Meta-Analysis

Inclusion criteria were defined for this meta-analysis. Specifically, we included observational studies such as case reports/studies and prospective and retrospective clinical studies with or without a control group (bone autograft). From these studies, we extrapolated the data relative to patients with long-bone nonunions treated with BMAC or BMSC alone or combined with scaffolds, having more than 18 years and no relevant comorbidities (i.e., congenital bone disorders, tumours, diabetes, and bone infections). Data relative to patients satisfying the inclusion criteria and presenting infected nonunions that were successfully treated before applying the cell-based therapy were included in this meta-analysis.

### 2.3. Study Selection

Three investigators (SL, SP, and ABL) independently reviewed the literature and classified the references based on the title and abstract. The eligible articles were further screened through the available full text, and the studies matching the inclusion criteria were selected. When a duplicate of published data was retrieved, we only considered the dataset reporting the complete dataset. Any disagreement was solved by discussion. In case a clinical study included both patients satisfying the eligibility criteria and patients that did not satisfy one or more inclusion criteria, the outcome data relative to the latter were excluded from the analysis. Studies reporting outcome data regarding eligible and noneligible patients with aggregated results were completely excluded. For instance, studies including patients with delayed unions and patients with bone nonunions that did not report the healing rate/time for each specific patient were excluded, since it was not possible to determine the healing rate only for patients with bone nonunions.

### 2.4. Data Extraction

Data extraction was performed by three investigators. Any disagreement was solved by discussion. The following data were extracted: type of treatment, number of included patients, age of patients, site and type of fracture, type and duration of bone nonunion, frequency of radiographic follow-up, healing rate at 6 and 12 months, failure rate at the last follow-up (i.e., variable time point depending on the study design) and reason, type of study, quality assessment, and reference. Treatments were classified into four categories: BMAC, BMAC combined with scaffold (BMAC/Scaffold), BMSC, and BMSC combined with scaffold (BMSC/Scaffold). Outcome data of different treatments included in the same article were individually considered and analysed according to the corresponding treatment category. The same approach was applied for studies reporting data from eligible and noneligible patients. For instance, in case a study that included data relative to patients with and without comorbidities, only the data relative to the latter were included in the meta-analysis.

### 2.5. Outcome(s)

1. The primary outcome was the bone healing rate investigating cell-based therapies (BMAC/Scaffold, BMSC/Scaffold) versus bone autograft as well as assessing the bone healing rate associated with four different types of cell-based therapies (BMAC, BMAC/Scaffold, BMSC, and BMSC/Scaffold) over a follow-up of 6 and 12 months
2. The secondary outcome was the correlation between the type and site of nonunion and the efficacy of the aforementioned treatments

### 2.6. Quality Assessment

As a measure of study quality, we selected and evaluated the following biases: (1) retrospective or prospective analysis and source of data (record bias), (2) relevance and definition of nonunion (i.e., lack of data regarding the duration, type, and site of nonunion) (reporting bias), (3) presence in the study of relevant confounding variables that could affect the clinical outcome (i.e., different types of nonunion, different sites of nonunion, different types of fixation, and different number of treatments) (relevant confounding factors bias), and (4) any missing outcome data (outcome reporting bias). Three investigators performed the quality assessment. Disagreements were resolved by consensus.

### 2.7. Statistical Analysis

For controlled studies, we evaluated the treatment effects based on the healing rate at 6 and 12 months as dichotomized outcomes, using the Risk Difference (RD) expressed as 95% confidence intervals (95% CIs). The outcome measures reported in the individual studies were combined by meta-analysis using random effect models, as described by DerSimonian and Laird [11], because a certain degree of heterogeneity of population and treatments would be expected among interventions.

Since the majority of the identified studies did not include any control group, we used the proportional meta-analysis to indirectly compare the different treatments along their CIs. The Effect Size (ES) represented the percentage of healing rate at 6 and 12 months with respect to the total number of patients included in each study per treatment group (BMAC, BMAC/Scaffold, BMSC, and BMSC/Scaffold). The forest plot presents specific proportions with 95% exact CIs for each study, the subgroup and overall pooled estimate with 95% Wald CIs, and the *I*^2^ statistic, which describes the percentage of total variation due to interstudy heterogeneity.

Statistical heterogeneity was assessed using the *I*^2^ statistic and assumed to be influential when the *I*^2^ was greater than 50% and *p* < 0.05 as statistically significant for the calculation of heterogeneity.

All the analyses were done with RevMan 5.3 [12] and STATA software version 15 using the metaprop command [13] as an adaptation of the metan programme developed by Harris et al. [14].

## 3. Results

### 3.1. Study Selection

**Figure 1:**
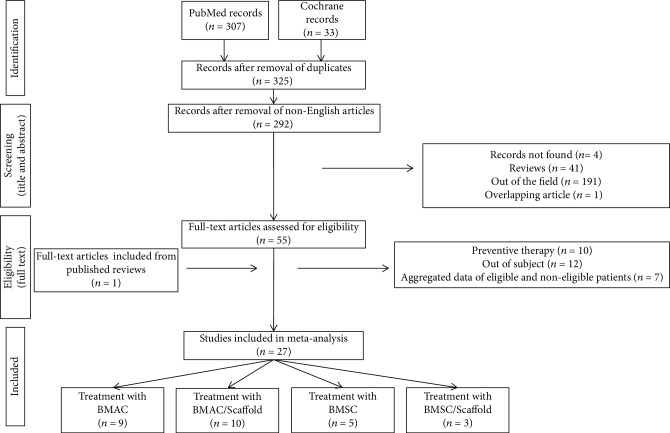
Flow diagram of the study selection process.

Based on the literature search strategy, 340 studies were found (307 in PubMed and 33 in Cochrane). Among them, 15 articles were excluded because they were doubly reported in the literature search. Of the remaining 325 records, 33 records were excluded because they were non-English articles. Other articles were also excluded for different reasons: 4 not available full text, 41 review articles, 1 overlapping study, and 191 articles not satisfying the inclusion criteria. Of the remaining 55 articles, after reading the full text, 29 were excluded for the following reasons: 10 papers describing a preventive therapy in the case of bone fracture, 12 not pertinent with the field of study, and 7 because only a part of patients satisfied all the inclusion criteria and it was not possible to extrapolate the healing rate of single patients. One study was retrieved from the bibliography of a review article. Overall, we finally included 27 studies meeting our eligibility criteria for the subsequent meta-analysis (Figure 1).

### 3.2. Features of the Studies and Quality Assessment

The studies included in the meta-analysis are reported in Table 1. About 70% of the analysed articles (19/27) concerned BMAC-based treatments; the remaining articles (8/27) investigated the efficacy of BMSC-based therapies. About half of the studies (13/27) combined BMAC or BMSC with a scaffold. Bone-derived (i.e., decellularized bone and bone chips) and ceramic scaffolds were the most used types of scaffolds. In total, the analysed studies included 347 patients treated for bone nonunions. Considering the different interventions, 163 patients were treated with BMAC, 153 with BMAC/Scaffold, 22 with BMSC, and 9 with BMSC/Scaffold. Patients' age ranged between 18 and 92 years. Almost half of the treated nonunions concerned the treatment of the tibia/fibula (52.9%), followed by the femur (32.3%), humerus/ulna (11.1%), and radius (3.7%). Only one study, including 50 patients, omitted to specify the number of treated femoral or tibial nonunions. For this reason, this study was not included in the calculation of the above described percentages. The type of fracture was described in 16 out of 27 studies (59.3%), with 76.6% of patients treated for closed fractures. Among the studies describing the treatment of open fractures, 3 reported the AO-OTA classification and the remaining reported the Gustilo and Anderson classification. None of the studies reported the defect size. The type of treated nonunion, i.e., atrophic or hypertrophic, was indicated in 14 articles (51.9%) and showed that most of the patients were treated for an atrophic nonunion (86.3%). The duration of nonunion ranged between 6 months and 9 years. Timing and frequency of radiographic follow-ups were very variable among studies. In 4 out of 27 studies, the latest follow-up was 6 months after treatment. Healing rate at 6 and 12 months was reported in 21 and 18 studies, respectively. Among the 15 articles reporting treatment failures at the last analysed follow-up (55.6%), only 6 provided a possible explanation, such as alcoholism, improper osteosynthesis, or bone loss. Adverse events were evaluated in most of the articles, but no side effects directly linked to the delivery of BMAC or BMSC were reported, suggesting that these treatments can be considered safe also in elders. Almost half of the studies were prospective (14/27). The remaining articles included 9 retrospective studies, 3 case studies, and 1 case report. Only 2 studies were controlled and included bone autograft as the control group. However, none of these studies was randomized.

The recorded bias was considered positive for retrospective and case studies/reports. Almost half of the articles presented this bias (15/27). This bias was attributed also to 2 prospective studies since it appeared that they gathered together a series of patients rather than pursuing a systematic and rational patient enrolment. We defined the reporting bias as an omission of relevant information regarding the characteristics of the treated nonunion, such as the type and the site of nonunion. This bias was attributed to 14 out of 27 articles (51.9%). Considering the high variability in the duration of nonunion, even within the same study, and other influencing factors, such as the type and site of nonunion and the type of fixation, almost all the studies were classified as biased by relevant confounding factors. Among the analysed studies, 2 out of 27 (7.4%) did not report the healing rate at the time points defined in the “materials and methods” of the study. Hence, these were classified as affected by outcome reporting bias. The frequency of the radiographic follow-up was not clearly defined in 11 articles (40.7%). Due to the lack of a clear follow-up plan, it was not possible to define if these records were biased or not by outcome reporting.

### 3.3. Bone Healing Rate at 6 Months of Follow-Up

**Figure 2:**
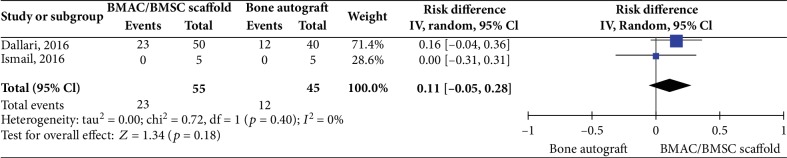
Bone healing rate at 6 months posttreatment for cell-based therapies vs. bone autograft.

Two controlled studies investigating cell-based therapies (BMAC/Scaffold or BMSC/Scaffold interventions) did not show any significant difference in bone healing rate versus the control intervention (bone autograft) (2 studies, 100 patients, RD 0.11 95% CI -0.05 to 0.28, *I*^2^ = 0%; *p* = 0.18, Figure 2).

**Figure 3:**
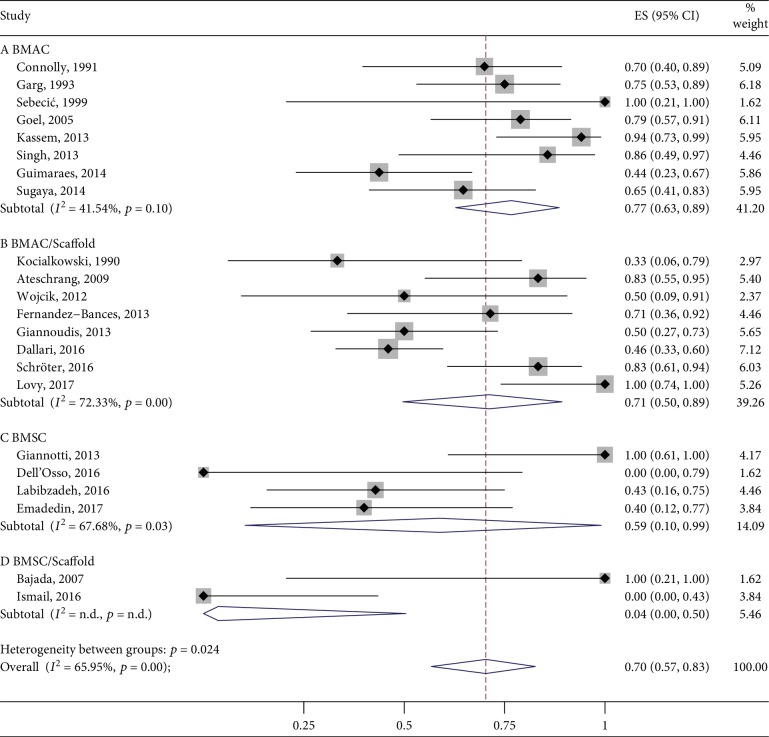
Bone healing rate at 6 months posttreatment.

Looking at the cohort studies lacking the control group, 5 studies did not report the outcome data as planned in their “materials and methods” section (18.5%). Of the remainder, the mean pooled proportion of bone healing rate in the BMAC group was 77% (95% CI 63%-89%, 107 cases, *n* = 8 studies) followed by BMAC/Scaffold with 71% (95% CI 50%-89%, 117 cases, *n* = 8), BMSC with 59% (95% CI 10%-99%, 19 cases, *n* = 4), and BMSC/Scaffold with 4% (95% CI 0%-50%, 6 cases, *n* = 2). The combined overlapped 95% CIs suggested a similar effect among the interventions, with the exception of the BMSC/Scaffold group that, however, was unrepresentative. Data are reported in Figure 3.

### 3.4. Bone Healing Rate at 12 Months of Follow-Up

**Figure 4:**
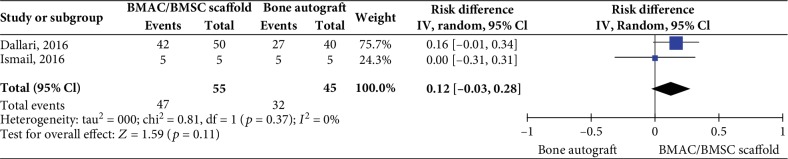
Bone healing rate at 12 months posttreatment for cell-based therapies vs. bone autograft.

Two controlled studies investigating BMAC/Scaffold or BMSC/Scaffold interventions did not show any significant difference in bone healing rate versus the control intervention (bone autograft) (2 studies, 100 patients, RD 0.12 95% CI -0.03 to 0.28, *I*^2^ = 0%; *p* = 0.11, Figure 4).

**Figure 5:**
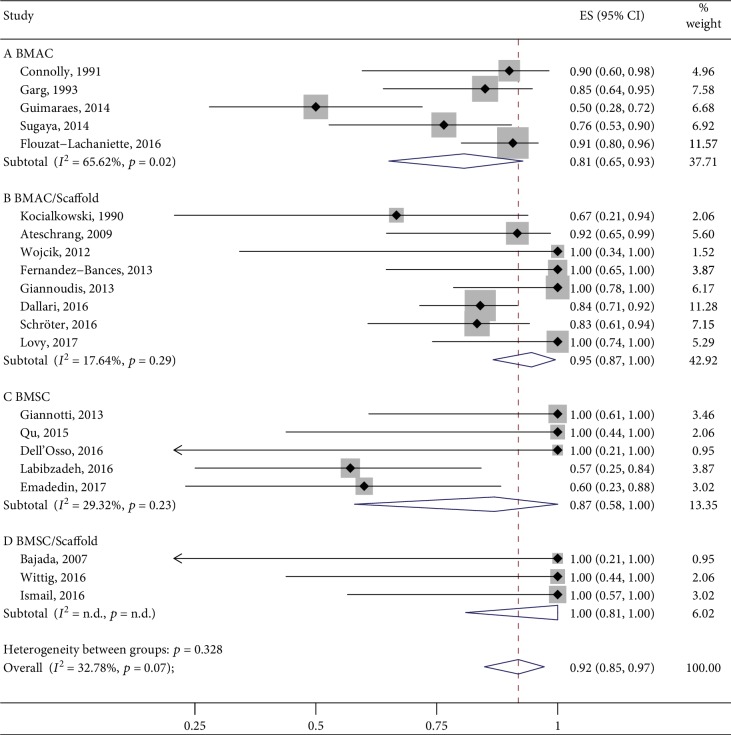
Bone healing rate at 12 months posttreatment.

Looking at the cohort studies lacking the control group, 2 studies did not report the outcome data as planned (4.7%). Of the remainder, the mean pooled proportion of bone healing in the BMSC/Scaffold treatment group was 100% (95% CI 81%-100%, 9 cases, *n* = 3) followed by BMAC/Scaffold with 95% (95% CI 87%-100%, 117 cases, *n* = 8), BMSC alone with 87% (95% CI 58%-100%, 22 cases, 7 = 5), and BMAC alone with 81% (95% CI 65%-93%, 117 cases, *n* = 5). The combined overlapped 95% CIs suggested similar effects among the four interventions studied. Data are reported in Figure 5.

## 4. Discussion

The standard management of nonunions is based on surgeries that may or may not involve the replacement of the fixation implants and the use of autografts. Nonunions represent a major indication for the clinical use of cell-based therapies. In fact, as indicated by the growing literature, BMSC and bone marrow derivatives have raised interest for the treatment of this disorder. In this systematic review, we noticed a positive trend in bone healing in favour of stem cell-based therapies as compared to bone autograft. However, it must be underlined that this difference was not statistically significant and data were weakly supported by a paucity of studies. In fact, the most represented study designs were single cohort studies. Among these, we found that BMAC alone or combined with scaffolds yielded bone healing at 6 months of follow-up in 77% and 71% of patients, respectively. Conversely, interventions with BMSC and BMSC/Scaffold showed an inferior healing rate 6 months after treatment (59% and 4%, respectively). The bone healing rate significantly increased for the BMAC/Scaffold group at 12 months of follow-up (95%), while it was only slightly increased when BMAC alone was used (81%). At this time point, the healing rate associated with BMSC-based treatments was even better than that yielded by the corresponding BMAC-based treatments (BMSC: 87%; BMSC/Scaffold: 100%). However, no definitive conclusion can be derived about the use of BMSC combined with a scaffold due to the retrieval of only 3 studies for a total number of 9 patients. Even in the absence of strong evidences coming from controlled studies, these preliminary results suggest that the use of bone scaffolds (demineralised bone, bioceramics, bone chips, etc.) in combination with cell-based therapies can be considered of great importance to locally deliver and engraft progenitor cells, while providing a structural support to the bone healing process.

In general, failures occurred mainly because of fracture instability, severe bone loss, or alcohol/drug addiction. We also speculated that one of the main causes of failure for cell-based treatment could be the presence of hypertrophic rather than atrophic nonunions which are usually not recommended for these kinds of orthobiologic approaches. Indeed, the efficacy of the cell-based therapies could be very limited in these cases where bone biological activity is retained compared to atrophic nonunions [42]. Moreover, hypertrophic nonunions are greatly affected by the fixation technique used to limit the micromovement of bone stumps, which may additionally complicate the situation. We considered these premises as particularly relevant to evaluate the efficacy of cell-based therapies in correlation with the type of nonunion. However, only half of the analysed articles reported the type of nonunion and the relative failure rate, which impeded us in investigating properly possible correlations between these two parameters. When treating long-bone nonunions, it is also important to keep in mind the type of injured bone. Indeed, leg and arm bones bear very different weights even in the same limb, which can significantly affect the clinical outcome. Albeit almost all the papers reported in which anatomical site the treatment was unsuccessful, the number of cases was still too low to clearly define a correlation between failure rate and fracture site. In the same way, it was not possible to investigate the correlation between the clinical outcome of each treatment and the type of fracture. Indeed, only about 50% of the studies declared whether the fracture was closed or open and/or provided a score in the case of open fractures. Additionally, in some studies where the fracture score was provided, the clinical outcome of each specific patient was not reported, thus impeding the correlation between these parameters.

Despite the mean age of treated patients being similar among the analysed studies, the age range was quite heterogeneous and widely distributed. Although the older age does not directly affect the healing outcomes of fracture nonunions [43], the quality of patients' mesenchymal stromal cells is known to be reduced in older patients [44–46]. According to this premise, the therapeutic efficacy of cell-based approaches should be weighted on the age of enrolled patients, similarly to metabolic and social habits that have been indicated as possible determinants of treatment failure in some of the analysed studies.

In this meta-analysis, we found that detailed reporting on failure rate and causes is often inconsistent. This implies the impossibility to clearly identify the association between known or possible confounding bias and treatment failure.

Based on all these considerations, we would like to highlight a very poor reporting of useful data regarding the patients' cohort and the clinical outcomes and subsequently stress the need to conduct studies in accordance with the international standards of reporting, as indicated by the EQUATOR network initiative and implemented by the STROBE guidelines for observational studies (http://www.equator-network.org/). Poor reporting in many cases also reflects a poor quality of conduct of these studies, explaining the mismatch between the amount of research spent in the last 20 years of *in vitro* and *in vivo* studies supporting the use of mesenchymal stromal cells in bone regeneration and the missing demonstration of their therapeutic efficacy for clinical practice. In fact, the present review retrieved only 2 controlled clinical trials comparing the efficacy of bone marrow-derived cell-based therapies to bone autograft, which represent the therapeutic gold standard [31, 41]. Anyway, these 2 studies were not randomized. Additionally, we noticed a huge variability in the procedures used in the analysed studies. For instance, both isolation and expansion protocols of BMSC and the protocols to obtain BMAC varied significantly among studies. Another variable, and often undefined, factor was the number of progenitor cells delivered either as BMAC or as expanded BMSCs. In fact, only few studies assessed the number of progenitor cells in BMAC [21–23, 28]. With regard to concerns on the use of expanded BMSC, we found studies delivering a variable number of BMSCs but none of these studies analysed the correlation between cell number and treatment efficacy [34, 36–38]. In this scenario, we believe that the recently approved EU-H2020-ORTHOUNION project will provide interesting insights. This project is focused on testing the efficacy of different doses of *in vitro* expanded BMSC loaded onto biomaterials in a transnational multicentre, controlled (autograft), and randomized clinical trial. Due to these specific features, the EU-H2020-ORTHOUNION represents a promising attempt to prove the efficacy of cell-based therapies for the treatment of nonunions in a standardized setup (EudraCT number 2015-000431-32) [47]. This study will enrol more than 100 patients, implementing a sample size calculation based on an accurate power analysis. Considering that among the analysed studies, 7 were case reports describing data obtained from 1 to 3 patients and all the studies evaluating expanded BMSC included less than 10 patients, the EU-H2020-ORTHOUNION project will represent a significant step forward and a positive example of an efficient collaboration among European clinicians.

## 5. Conclusions

Overall, the data obtained in this meta-analysis should be interpreted with caution. Indeed, the heterogeneity of the studies along with limited reporting and nonsystematic study design makes difficult to draw clear and definitive recommendations regarding the best approach to heal bone nonunions. Clinical trials recapitulating a more accurate planning and data collection should be conducted in the future to obtain a reliable demonstration of the efficacy of cell-based therapies, the importance of using bone scaffolds to promote the engraftment of implanted progenitor cells, and the superiority of BMAC versus expanded BMSCs.

## Figures and Tables

**Table 1:** Studies selected based on inclusion and exclusion criteria. Fracture grading is reporting based either on Gustilo and Anderson (G) or on AO-OTA (OTA) classification scale.

Treatment	*n* subjects Age range (years)	Site of fracture	Type of fracture	Type of nonunion	Duration of nonunion	Frequency of radiographic follow-up	Healing rate at 6 months (healed/total)	Healing rate at 12 months (healed/total)	Failure rate at last follow-up Failure reason(s)	Type of study	Record bias	Reporting bias	Relevant confounding factor bias	Outcome reporting bias	Ref.
BMAC	10 subjects 18-82	10 tibias	1 closed 6 GII 3 GIII	n.d.	7-36 months	n.d.	7/10	9/10	10% n.d.	Prospective	No	Yes	Yes	—	[15]

BMAC	20 subjects 18-65	15 tibias 3 humeri 2 ulnae	10 closed 6 GI 2 GII 2 GIII	n.d.	6-18 months	Monthly	15/20	17/20	15% Fractures with bone loss and ulnar fracture	Prospective	No	Yes	Yes	No	[16]

BMAC	1 subject 44	1 tibia	1 GIII	n.d.	6 months	Every 6 weeks (until 6 months)	1/1	n.d.	0%	Case study	Yes	Yes	No	No	[17]

BMAC	19 subjects 24-60	19 tibias	n.d.	9 atrophic 10 hypertrophic	6-36 months	Every 4-6 weeks (until 6 months)	15/19	n.d.	21% n.d.	Prospective	No	No	Yes	No	[18]

BMAC	17 subjects 25-66	5 femurs 10 tibias 2 ulnae	17 closed	n.d.	6-24 months	Monthly (until 6 months)	16/17	n.d.	6% Improper osteosynthesis	Prospective	No	Yes	Yes	No	[19]

BMAC	7 subjects 25-66	2 femurs 2 humeri 5 ulnae	7 closed	7 atrophic	7-53 months	n.d. (until 6 months)	6/7	n.d.	10% Alcoholism	Retrospective	Yes	No	Yes	—	[20]

BMAC	16 subjects 19-59	16 femurs	12 closed 3 GI 1 GII	7 atrophic 9 oligotrophic	9-97 months	1.5, 4, 6, and 12 months	7/16	8/16	10% Alcoholism	Prospective	No	No	Yes	No	[21]

BMAC	17 subjects 22-80	10 femurs 5 tibias 1 ulna 1 humerus	12 closed 5 open	17 atrophic	9-79 months	1, 2, 3, 6, 9, and 12 months	11/17	13/17	23.5% n.d.	Retrospective	Yes	No	Yes	No	[22]

BMAC	54 subjects 22-64	54 tibias	54 closed	n.d.	n.d.	n.d.	n.d.	49/54	9.25% n.d.	Retrospective	Yes	Yes	Yes	—	[23]

BMAC/Scaffold (porous ceramic mixed with collagen)	3 subjects 26-60	1 femur 1 tibia 1 ulna	n.d.	n.d.	7-12 months	1.5, 3, 6, and 12 months	1/3	2/3.	0%	Prospective	Yes	Yes	Yes	Yes	[24]

BMAC/Scaffold (DBM)	15 subjects 22-87	3 femurs 10 tibias 2 humeri	n.d.	n.d.	n.d.	n.d.	n.d.	n.d.	40% n.d.	Prospective	Yes	Yes	Yes	—	[25]

BMAC/Scaffold (allogeneic bone graft)	12 subjects 19-68	12 tibias	2 closed 1 GI 3 GII 6 GIII	n.d.	>6 months	n.d.	10/12	11/12	8.33% n.d.	Prospective	No	Yes	Yes	—	[26]

BMAC/Scaffold (frozen cancellous bone)	2 subjects 22 and 38	2 humeri	n.d.	n.d.	>6 months	n.d.	1/2	2/2	0%	Retrospective	Yes	Yes	Yes	—	[27]

BMAC/Scaffold (cancellous bone)	7 subjects 26-70	3 femurs 3 tibias 1 ulna	n.d.	n.d.	>6 months	Monthly up to 12 months Yearly afterwards	5/7	7/7	0%	Prospective	No	Yes	Yes	No	[28]

BMAC/Scaffold (autologous bone chips)	14 subjects 33-92	14 femurs	9 OTA32-A2-1 3 OTA32-B3-1 2 OTA31-A2-3	14 atrophic	16-48 months	1.5, 3, 4, 5, 6, 8, 12, and 18 months or until union	7/14	14/14	0%	Retrospective	Yes	No	Yes	No	[29]

BMAC/Scaffold (DBM)	19 subjects 20-73	1 femur 8 tibias 3 humeri 3 radii 2 ulnae 2 metatarsi	n.d.	19 atrophic	>9 months	n.d.	n.d.	n.d.	21% n.d.	Prospective	No	No	Yes	—	[30]

BMAC/Scaffold (autologous bone chips+PRP)	50 subjects 18-76.7	n.d. femur n.d. tibia	n.d.	39 atrophic 11 hypertrophic	>6-9 months	1.5, 3,6, 12, and 24 months	23/50	42/50	4.88% Bone loss and failure of fixation device	Retrospective Controlled	Yes	Yes	Yes	No	[31]

BMAC/Scaffold (allogeneic cancellous bone)	18 subjects 19-81	18 femurs	17 closed 1 GIII	n.d.	6 months	Monthly	15/18	15/18	16.7% Impaired bone vascularity	Prospective	No	Yes	Yes	No	[32]

BMAC/Scaffold (DBM)	11 subjects 55-79	11 femurs	n.d.	n.d.	>6 months	0.5, 1.5, 3, 6, 9, and 12 months	11/11	11/11	0%	Retrospective	Yes	Yes	Yes	No	[33]

BMSC	6 subjects 18-73	3 humeri 1 ulna 1 radius 1 ulna and radius	1 OTA12-A1 1 OTA12-B3 1 OTA12-C1 1 OTA22-A1 1 OTA22-A2 1 OTA22-C3	6 atrophic	n.d.	n.d.	6/6	6/6	0%	Retrospective	Yes	No	Yes	—	[34]

BMSC	3 subjects 19-44	1 femur 1 humerus 1 forearm	n.d.	n.d.	19-39 months	3, 6, and 12 months	n.d.	3/3	0%	Retrospective	Yes	Yes	Yes	Yes	[35]

BMSC	1 subject 47	1 ulna	1 OTA22-A1-1	1 atrophic	6 months	n.d.	0/1	1/1	0%	Case study	Yes	No	No	—	[36]

BMSC	7 subjects 26-61	4 femurs 3 tibias	3 closed 4 open	7 atrophic	8-96 months	1, 3, 6, and 12 months	3/7	4/7	42.8% n.d.	Prospective	No	No	Yes	No	[37]

BMSC	5 subjects 23-55	3 femurs 2 tibias	5 closed	5 atrophic	7-72 months	1, 3, 6, and 12 months	2/5	3/5	40% n.d.	Prospective	No	No	Yes	No	[38]

BMSC/Scaffold (CaSO_4_ pellets)	1 subject 34	1 tibia	1 closed	1 hypertrophic	9 years	n.d.	1/1	1/1	0%	Case study	Yes	No	No	—	[39]

BMSC/Scaffold (collagen microspheres)	3 subjects 27-81	1 femur 2 tibias	n.d.	3 atrophic	12-24 months	n.d.	n.d.	3/3	0%	Case reports	Yes	No	Yes	—	[40]

BMSC/Scaffold (HA granules)	5 subjects 18-37	3 femurs 1 tibia 1 humerus	n.d.	5 atrophic	37.2 months	Monthly up to 12 months	0/5	5/5	0%	Prospective Controlled	No	No	Yes	No	[41]

n.d.: nondescribed.

## Supplementary Materials

Supplementary Materials Appendix S1: search strategy including the MeSH terms used for the literature search. Appendix S2: table including eligible studies that were excluded since they reported aggregated data for eligible and noneligible patients.
