# Supplementary material for: Bone Marrow-Derived Cell Therapies to Heal Long-Bone Nonunions: A Systematic Review and Meta-Analysis—Which Is the Best Available Treatment?
Source: Stem Cells Int. 2019 Dec 27;2019:3715964. doi: 10.1155/2019/3715964 (PMC6948316; doi:10.1155/2019/3715964)
Supplement: Supplementary Materials — Appendix S1: search strategy including the MeSH terms used for the literature search. Appendix S2: table including eligible studies that were excluded since they reported aggregated data for eligible and noneligible patients. [file 3715964.f1.doc]

## Supplementary Materials

**Appendix S1. Search strategy**

MeSH terms used included:

1. Human
2. Non-union OR Nonunion OR Pseudoarthrosis OR delayed union OR Ununited OR atrophic bone
3. Stem cell OR stromal cell OR cell therapy OR mesenchymal cell OR bone marrow aspirate OR bone marrow aspirate concentrate OR bone marrow concentrate OR MSC OR BMSC OR BMAC OR cell transplantation OR Bone Marrow OR BMC
4. 1 AND 2 AND 3

**Appendix S2. Table including eligible studies that were excluded since they did not report single data for eligible and non-eligible patients**

| **Treatment** | ***n* subjects**  **Age range**  ***[years]*** | **Lesion site** | **Data excluded from meta-analysis and reason** | **Reference** |
| --- | --- | --- | --- | --- |
| BMAC | 60 subjects  18-78 | Tibia | Includes diabetic patients and does not report single data | Hernigou *et al.*  (2005)  doi: 10.2106/JBJS.D.02215 |
| BMAC | 11 subjects  24-51 | Tibia | Includes both delayed union and nonunions but does not report single data | Braly *et al.*  (2013)  doi: 10.1097/BOT.0b013e31828bf077 |
| BMAC | 45 subjects  15-85 | Femur, tibia, humerus | Includes diabetic patients and does not report single data | Gross *et al.*  (2015)  doi: 10.3233/BME-141235 |
| BMAC/Scaffold  (DBM ± BMP2) | 49 subjects  19-93 | Femur, tibia, humerus | Includes both delayed union and nonunions but does not report single data | Desai *et al.*  (2015)  doi: 10.1007/s11420-015-9432-1 |
| BMAC/Scaffold  (autologous bone chips) | 64 subjects  17-83 | Femur, tibia, humerus, radio, clavicle | Includes diabetic patients and does not report single data | Giannoudis *et al.*  (2015)  doi: 10.1016/S0020-1383(15)30055-3 |
| BMSC/Scaffold (bioceramic) | 26 subjects  n.d. | Femur, tibia, humerus | Includes both delayed union and nonunions but does not report single data | Gomez-Barrena *et al.*  (2018)  doi: 10.1016/j.biomaterials.2018.03.033 |
| BMSC/Scaffold  (bone chips + PRP) | 1 subject  32 | Femur | Patient subjected to a new surgical procedure due to a new fracture in the same limb | Dallari *et al.*  (2012)  doi: 10.1007/s00167-011-1790-8 |
